# Considerations in Neuromuscular Blockade in the ICU: A Case Report and Review of the Literature

**DOI:** 10.1155/2020/8780979

**Published:** 2020-03-07

**Authors:** Jessica D. Workum, Stephanie H. V. Janssen, Hugo R. W. Touw

**Affiliations:** ^1^Department of Intensive Care, Radboud University Medical Center, Geert Grooteplein Zuid 10, 6525 GA, Nijmegen, Netherlands; ^2^Department of Clinical Pharmacology, Radboud University Medical Center, Geert Grooteplein Zuid 10, 6525 GA, Nijmegen, Netherlands; ^3^Department of Anaesthesiology, Radboud University Medical Center, Geert Grooteplein Zuid 10, 6525 GA, Nijmegen, Netherlands

## Abstract

Neuromuscular blocking agents are regularly used in the intensive care unit (ICU) to facilitate mechanical ventilation in patients with acute respiratory distress syndrome and patient-ventilator dyssynchronies. However, prolonged neuromuscular blockade is associated with adverse effects like ICU-acquired weakness. Residual neuromuscular blockade is, however, not routinely monitored in the intensive care unit, and as such, this phenomenon might be unrecognized and underreported. We report a case in which an unusual prolonged effect of neuromuscular blockade was seen after cessation of the drug, which illustrates the complexity of neuromuscular blockade in the ICU. We advocate for the use of train-of-four measurements in the ICU, recommend to choose cisatracurium over rocuronium in critically ill patients due to their pharmacokinetics when continuous neuromuscular blockade is considered, and propose a subsequent strategy once the choice has been made to start neuromuscular blockade.

## 1. Introduction

Neuromuscular blocking agents (NMBAs) are used in the intensive care unit (ICU) for various indications, for instance, to facilitate intubation in the treatment of status asthmaticus and to facilitate mechanical ventilation in patients with acute respiratory distress syndrome (ARDS). The ACURASYS trial by Papazian et al. [1] found a beneficial effect of continuous neuromuscular blockade on the mortality of ARDS patients. This has led to an increase in the use of continuous NMBAs in the ICU, as the current guideline for sustained neuromuscular blockade in the adult critically ill patient now recommends starting continuous NMBAs early in ARDS [2]. In contrary to the ACURASYS trial, the recently published ROSE trial found no reduction in mortality with early continuous neuromuscular blockade in ARDS [3]. Furthermore, it was associated with an increase in ICU-acquired weakness and serious adverse cardiovascular events. Neuromuscular blockade should, therefore, be used cautiously and only in selected individuals in which other strategies have failed.

Neuromuscular blockade can have residual effects. Residual neuromuscular blockade can cause postoperative pulmonary complications [4], ranging from a reduced ability to swallow by a loss of coordination of tongue and throat musculature, which carries a higher risk of aspiration [5, 6], to general muscle weakness, loss of ventilatory hypoxic drive, atelectasis, and airway obstruction. An additional important complication is awareness. These complications can lead to an increased length of hospital stay with a threefold higher chance to be admitted to the ICU [7]. In the critically ill patients, the consequences of residual neuromuscular blockade have not been investigated; however, it is reasonable to assume a risk similar to prolonged infusion.

The degree of (residual) neuromuscular blockade can be determined by measuring the neuromuscular response on peripheral nerve stimulation, for instance, with a train-of-four (TOF) measurement. During TOF stimulation, four stimuli are delivered at 0.5 second intervals, and the response of the innervated muscle is assessed. With an increasing depth of neuromuscular blockade, this response (twitches) decreases in force. When there is no response on TOF stimulation, a deep neuromuscular blockade may be present, the intensity of which can be measured using the posttetanic count (PTC). During a PTC measurement, a 5-second tetanic stimulus of 50 Hz is administered, followed by a series of single twitch stimuli delivered at 1 Hz for 20 seconds. A response to this stimulation will be seen in the early stages of recovery [8]. Despite the frequent use of NMBAs in the ICU, it is not yet common practice to measure TOF or PTC in the critically ill and as such, residual neuromuscular blockade might be unrecognized and underreported.

We present a case that illustrates the complexity of continuous neuromuscular blockade in a critically ill patient. Furthermore, we review the literature and pharmacokinetics of neuromuscular blockade in the ICU and propose a subsequent strategy, once the choice has been made to start neuromuscular blockade.

## 2. Case Report

A 76-year-old woman was admitted to our ICU with pyogenic spondylodiscitis, due to *Staphylococcus aureus* bacteremia with unknown focus, complicated by an epidural abscess for which she received a laminectomy with subsequent drainage of the abscess. Her medical history consisted of a coronary artery bypass graft and hypertension. In the days following surgery, she developed ventilator-acquired pneumonia (VAP) with difficult mechanical ventilation, as expressed by patient-ventilator dyssynchronies like reverse triggering. As deep sedation with midazolam, sufentanyl, and propofol showed no improvement, it was decided to start continuous neuromuscular blockade with rocuronium. Though this did improve the quality of ventilation, we did find extensive residual effect of the neuromuscular blockade.

After 48 hours of continuous infusion, rocuronium was ceased. To rule out any form of residual neuromuscular blockade, TOF and PTC measurements were performed 5 hours after cessation of the rocuronium by stimulating the ulnar nerve. TOF response was zero, and no motor response could be provoked from the PTC stimulation either. It was concluded that there was still residual neuromuscular blockade, and therefore sedation was continued.

The next day, 22 hours after cessation of the rocuronium, both measurements were performed again under unchanged conditions. The results were similar: TOF response remained zero, and there was no response on PTC stimulation.

Approximately 60 hours after cessation of the continuous rocuronium infusion, the TOF remained immeasurably low, but this time, PTC stimulation gave a clear motor response that consisted of a few twitches with visible fading. We, therefore, administered 500 mg sugammadex intravenously split into two doses to antagonize rocuronium, after which the TOF ratio subsequently rose to 94 percent.

The following morning, the TOF ratio has fallen to 4 percent again without intervention due to recurarization. The measurement was repeated 5 hours later, and it had risen to 92 percent without intervention, upon which sedation was stopped successfully.

## 3. Discussion

### 3.1. Nondepolarizing Neuromuscular Blocking Agents

Nondepolarizing NMBAs bind to the alpha-subunit of the skeletal muscle postsynaptic nicotinic receptor, preventing acetylcholine from binding to the receptor and depolarizing the cell and thus inhibiting muscular contraction. They can be divided into two classes: the benzylisoquinolinium (e.g., atracurium and cisatracurium) and the aminosteroid compounds (e.g., rocuronium, vecuronium, and pancuronium). In the ICU, aminosteroid compounds are more commonly used than their benzylisoquinolinium counterparts, based on familiarity of the physician with the drug [9]. Unlike benzylisoquinolium NMBAs, aminosteroid NMBAs can be antagonized with sugammadex, a selective binding agent with high affinity. Sugammadex encapsulates the aminosteroid NMBA, negating its effect [10].

### 3.2. Pharmacokinetics of Rocuronium

Like all neuromuscular blockers, rocuronium is administered intravenously, as it is not effective orally. It is an aminosteroid compound which rapidly distributes throughout the body, following a three compartment model. The rapid distribution half-life is 1 to 2 minutes, whereas the slower distribution half-life is 14–18 minutes. It has a volume of distribution of 0.21–0.27 l/kg in the adult population, which decreases slightly in the elderly population. Protein binding is approximately 30%. Rocuronium is partially metabolized to the active metabolite 17-desacetyl-rocuronium, which is approximately 20 times less potent than rocuronium itself. Elimination half-life varies between 60 and 90 minutes. The primary route for excretion is biliary, whereas 33–47% of rocuronium is excreted unchanged in urine.

### 3.3. Pharmacokinetics of Cisatracurium

Cisatracurium is a benzylisoquinolinium compound. It is an isomer of atracurium with more favorable characteristics: it is four times more potent, does not cause histamine release, and has no direct cardiovascular effects. It has a shorter onset, with its peak within 1-2 minutes. It has a volume of distribution of 0.145 l/kg, and it is mainly metabolized through Hofmann elimination, with a plasma elimination half-life of 22 minutes. Renal and hepatic metabolism is negligible; however, the inactive metabolites are excreted renally. With 40–90 minutes, its duration of action is slightly longer than atracurium.

### 3.4. Train-of-Four in the Critically Ill

Currently, the guideline for sustained neuromuscular blockade in the adult critically ill patient recommends against monitoring the depth of neuromuscular blockade using TOF alone, but it suggests to use it in conjunction with clinical assessment [2]. Clinical assessment proves, however, to be an unreliable method for assessing the depth of neuromuscular blockade when compared to TOF, even in the critically ill [11].

It is not clear yet if the depth of neuromuscular blockade in patients with ICU-acquired weakness can be adequately measured with peripheral nerve stimulation [12]. TOF measurements are lower in patients with ICUAW than in those without. Results should, therefore, be interpreted with great care in this group of patients.

### 3.5. Neuromuscular Blockade in the Critically Ill

Though several sources have indicated that renal insufficiency does not impair rocuronium clearance [13, 14], clinical studies have shown prolonged neuromuscular effects of rocuronium in patients with renal failure [15, 16], though none come close to our patient. Liver disease affects rocuronium volume of distribution and clearance, increasing the duration of its effect [17, 18].

A study performed by Circeo et al. [19] showed a profound prolonged effect of rocuronium after continuous infusion in critically ill patients with multiple organ failure (MOF) compared to single organ failure. Mean time to recovery, as measured with the train-of-four, was 599 minutes for the MOF group versus 230 minutes in the non-MOF group. Furthermore, the MOF group required only 40% of the amount of rocuronium used non-MOF group (0.2 mg/kg/hour versus 0.5 mg/kg/hour).

It is, therefore, postulated that the prolonged effect of rocuronium in our patient is multifactorial. She had received a continuous dose of 0.3 mg/kg/hour twice the recommended maintenance dose of 0.1–0.15 mg/kg/hour. Her volume of distribution would have been increased as a result of excessive fluid administration due to prior shock as well as profound hypoalbuminemia (13 g/L, normal range 35–45 g/L), causing a prolonged distribution half-life. She showed signs of hepatic injury, with slight increases in transaminase levels (ALT 49 U/L and AST 184 U/L), elevated yGT (417 U/L), AF (319 U/L) and bilirubin levels (direct 43 umol/L, total 44 umol/L), attributed to high dose flucloxacillin as liver and biliary pathology was excluded on ultrasound, but no signs of liver failure as her glucose levels, platelet count, and coagulation panel were normal. And she had profound renal impairment (GFR <10 ml/min) for which continuous renal replacement therapy was started. Both could contribute to a decrease in rocuronium clearance, causing a prolonged clinical effect.

The administration of sugammadex and its subsequent reversal of neuromuscular blockade confirmed our suspicion of residual neuromuscular blockade. As sugammadex is predominantly renally cleared, its half-life is increased in renal dysfunction, possibly causing rocuronium-sugammadex complexes to circulate longer in the system. However, these complexes have a very low dissociation rate, so there is little to no concern of recurarization. In addition, the reversal rate is as rapid and effective as in patients with normal renal function. [20].

## 4. Conclusion

This case illustrates the complex pharmacokinetics of rocuronium in the critically ill, especially when administered as a continuous infusion, with an unexpectedly prolonged clinical effect. Combined with the recent findings of the ROSE trial, we strongly advise to exercise restrained use of continuous neuromuscular blockade in the ICU in general and advise to consider using intermittent boluses instead. If, however, the choice has been made to start continuous neuromuscular blockade, we strongly advise to monitor TOF levels during continuous infusion and after cessation of the drug. We furthermore advise to choose cisatracurium over a steroid-based NMBA like rocuronium in these situations, due to their more favorable pharmacokinetics in critically ill patients.

### 4.1. Proposed Strategy and Recommendations

For rapid sequence induction, we recommend a steroid-based NMBA like rocuronium, due to its rapid onset and the possibility to antagonize its effects with sugammadex.

If continuous neuromuscular blockade is considered, then

(1) Consider other strategies
  - Deeper sedation
  - Optimizing patient-ventilator synchrony
  - Others
(2) Consider intermittent boluses instead
(3) Consider your patient: if multiple organ failure, then
  - Monitor the effect with TOF
  - Choose cisatracurium over an aminosteroid NMBA
(4) Consider monitoring TOF levels in all patients
(5) If prolonged effects are observed after cessation of the drug, consider antagonizing with sugammadex if an aminosteroid (rocuronium, vecuronium, or pancuronium) was chosen. Mind that due to their distribution, this may need to be repeated.
